# [^89^Zr]Zr-PSMA-617 PET/CT in a Patient with Biochemical Recurrence of Prostate Cancer and Prior Indetermined Findings on [^18^F]PSMA-1007 Imaging

**DOI:** 10.3390/diagnostics14202321

**Published:** 2024-10-18

**Authors:** Moritz B. Bastian, Caroline Burgard, Arne Blickle, Tilman Speicher, Samer Ezziddin, Florian Rosar

**Affiliations:** Department of Nuclear Medicine, Saarland University, 66421 Homburg, Germany; caroline.burgard@uks.eu (C.B.); arne.blickle@uni-saarland.de (A.B.); tilman.speicher@uks.eu (T.S.) samer.ezziddin@uks.eu (S.E.); florian.rosar@uks.eu (F.R.)

**Keywords:** prostate cancer, PSMA, PET/CT, ^89^Zr, ^18^F, PSMA-1007

## Abstract

We report a case of a 79-year-old male patient with a history of radical prostatectomy for prostate cancer. The patient presented with biochemical reoccurrence; however, previous conventional PSMA PET/CT using [^18^F]PSMA-1007 showed two indetermined findings with low uptake in the right iliac lymph nodes. Further MRI evaluation provided no additional information. A recently introduced PSMA tracer, [^89^Zr]Zr-PSMA-617 (half-life: 3.3 days), was administered in an attempt to confirm the diagnosis and aid in potential radiation planning. [^89^Zr]Zr-PSMA-617 PET/CT clearly revealed the previously indetermined right iliac lymph nodes as definitely metastatic and also identified additional lymph node metastases that were undetected in prior scans. This case highlights the potential superior sensitivity of [^89^Zr]Zr-PSMA-617 PET/CT in detecting recurrent disease, especially in unclear settings of [^18^F]PSMA-1007 PET/CT and demonstrates its potential for guiding targeted radiation therapy with curative intent.

In recent years, PSMA-PET/CT with [^18^F]PSMA-1007, besides [^68^Ga]Ga-PSMA-11, revolutionized PET imaging and imaging-guided therapy of prostate cancer patients with biochemical recurrence [1,2,3,4,5,6]. However, in some cases, no clear imaging findings are observed (Figure 1), which can result in missed opportunities for curative, personalized treatments. Recently, PSMA PET/CT with long-lived ^89^Zr (half-life: 3.3 d) has shown promise in detecting lesions that are not visible with conventional [^68^Ga]Ga-PSMA-11 PET/CT [7,8]. These studies have demonstrated that BCR can be successfully localized at low PSA levels, identifying local recurrences and lymph node or bone metastases in most patients. This case (Figure.1) highlights the superior sensitivity of [^89^Zr]Zr-PSMA-617 PET/CT even in the case of prior [^18^F]PSMA-1007 PET/CT, although [^18^F]PSMA-1007 is also reported to be superior to [^68^Ga]Ga-PSMA-11 PET/CT for pelvic soft tissue findings due to its tracer kinetics [9]. Furthermore, [^68^Ga]Ga-PSMA-11 PET/CT has a low sensitivity for detecting small lymph node metastases [10]. The superior sensitivity of [^89^Zr]Zr-PSMA-617 in this case is mainly attributed to the longer half-life of ^89^Zr (3.3 days), which allows for delayed imaging. This results in a higher tumor-to-background ratio (such as the tumor-to-liver ratio) over time, potentially leading to more accurate visual detection of metastatic lesions. In contrast, [^18^F]PSMA-1007, with its shorter half-life (110 min), does not enable delayed imaging. The clinical significance of this case lies in the fact that [^89^Zr]Zr-PSMA-617 PET/CT provided definitive evidence of metastatic lymph node involvement, which had been indeterminate on [^18^F]PSMA-1007 PET/CT and MRI. This allowed for more accurate radiation planning with a curative intent, which might not have been feasible with the previous imaging results. While [^89^Zr]Zr-PSMA-617 PET/CT may present higher upfront costs and limited availability compared to other tracers, its ability to provide more definitive imaging results in cases where conventional tracers yield indeterminate findings can potentially prevent mismanagement or the need for repeated investigations. Altogether, this highlights the potential of [^89^Zr]Zr-PSMA-617 to improve clinical decision-making in prostate cancer recurrence, offering personalized, potentially curative treatment options. Therefore, clinicians should consider using [^89^Zr]Zr-PSMA-617 in similar cases to support individualized, potentially curative treatments, and future studies are recommended, ideally in a prospective setting, when conventional [^18^F]PSMA-1007 PET/CT has failed or is indeterminate. The primary endpoints of future studies should focus on the sensitivity and specificity of [^89^Zr]Zr-PSMA-617 in detecting metastatic lesions and its impact on clinical decision-making, such as radiation therapy planning.

## Figures and Tables

**Figure 1:**
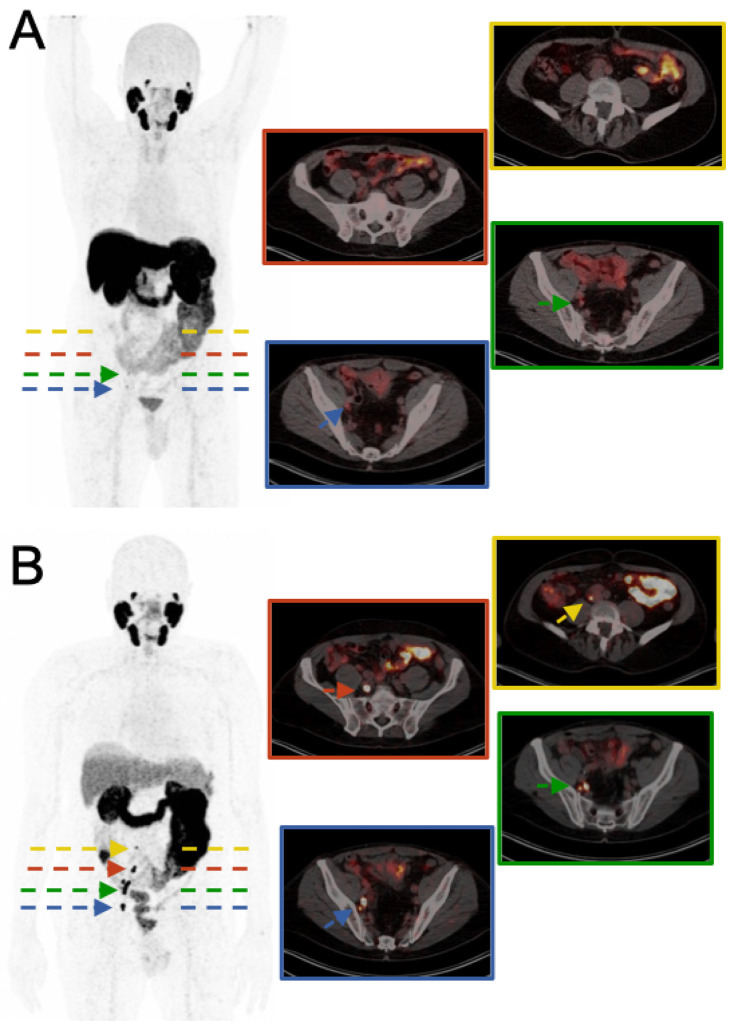
A 79-year-old male patient with a 23-year history of radical prostatectomy for prostate cancer was referred to our clinic due to biochemical recurrence (BCR), with rising prostate-specific antigen (PSA) levels reaching 0.84 ng/mL. To localize recurrence, we performed a [^18^F]PSMA-1007 PET/CT (**A**), which revealed two indetermined findings with low uptake (SUV_max_ up to 6.4, tumor-to-liver ratio (TLR) 0.39, SUV_mean_ liver 16.6) in right iliac lymph nodes potentially suspicious for lymph node metastases. For further evaluation, an MRI of the pelvic region was conducted, but provided no additional constructive information. In an attempt to confirm the diagnosis and aid in potential radiation planning, we opted to administer a novel PSMA PET tracer using long-lived ^89^Zr (half-life: 3.3 days), with PET/CT imaging performed 48 h after injection of 147 MBq [^89^Zr]Zr-PSMA-617 (**B**). The [^89^Zr]Zr-PSMA-617 PET/CT revealed the two right iliac lymph nodes as definitely metastatic with intense uptake (SUV_max_ up to 49.5, TLR 12.69, SUV_mean_ liver 3.9) and identified additional pelvic and retroperitoneal lymph node metastases (SUV_max_ up to 35.1, TLR 9.0, SUV_mean_ liver 3.9) that had not been detected on the prior scans. In this case, stereotactic body radiation therapy (SBRT) was the planned treatment following the confirmation of metastatic lymph node involvement.

## Data Availability

The datasets used and analyzed in this paper are available from the corresponding author on reasonable request.
